# Harmonization of Cognitive Function in Longitudinal Integrative Data Analysis: Results from two Chinese datasets

**DOI:** 10.1093/geroni/igaf122.2294

**Published:** 2025-12-31

**Authors:** Chang Yu

**Affiliations:** University at Buffalo, SUNY, Getzville, New York, United States

## Abstract

**Objective:**

Integrating data from multiple longitudinal studies is crucial for understanding cognitive aging across different population groups. However, common harmonization methods, such as z-scores or percentiles, often fail to account for differences in measurement properties and sample demographics, potentially introducing bias. This study applies Item Response Theory (IRT) to harmonizing general and domain-specific cognitive function across two major Chinese national longitudinal aging studies. By developing a harmonized framework, this study aims to enhance comparability while ensuring reliability and validity in pooled analyses.

**Method:**

The sample consists of individuals who completed at least one wave of cognitive testing from two major datasets. The Chinese Longitudinal Healthy Longevity Survey (CLHLS) (N = 38,760; 1998–2018; 8 waves) primarily focuses on older adults (65+), while the China Health and Retirement Longitudinal Study (CHARLS) (N = 19,150; 2011–2018; 4 waves) includes adults aged 45 and older. This study applies factor analysis to identify common latent cognitive constructs and employs IRT-based scale linking to align cognitive scores. Differential Item Functioning (DIF) analysis is conducted to examine variations in different datasets.

**Results:**

Preliminary findings suggest that factor score harmonization improves measurement alignment compared to traditional standardization techniques. However, differences in item properties and demographic distributions pose challenges, requiring further refinement of linking procedures.

**Discussion:**

This study advances longitudinal integrative data analysis in cognitive aging research, demonstrating the feasibility of factor score-based harmonization while highlighting potential limitations. Future work will refine linking approaches and assess how harmonization impacts cognitive trajectory analyses.

